# Follow-up of women with inadequate Pap smears: a prospective cohort study: Acompanhamento de mulheres com esfregaços de Papanicolaou inadequados: um estudo prospectivo de coorte

**DOI:** 10.1590/1516-3180.2013.7070004

**Published:** 2014-11-28

**Authors:** Fanny López-Alegría, Dino Roberto Soares De Lorenzi, Orlando Quezada Poblete

**Affiliations:** I PhD. Associate Professor, School of Nursing, Universidad Andres Bello, Santiago, Chile.; II MD, PhD. Adjunct Professor, Department of Obstetrics and Gynecology, University of Caxias do Sul, Brazil.; III MT. Medical Technologist, Cytology Laboratory, Complejo Asistencial Barros Luco, Santiago, Chile.

**Keywords:** Cell biology, Vaginal smears, Uterine cervical neoplasms, Health planning guidelines, Follow-up studies

## Abstract

**CONTEXT AND OBJECTIVE:**

Inadequate Pap smears do not provide satisfactory cell samples for evaluation, thus making it more difficult to detect cervical cytological abnormalities. The objective of this study was to determine the cytological and histological follow-up results from women with inadequate smear reports in primary healthcare centers in Santiago, Chile 2010-2011.

**DESIGN AND SETTING:**

Prospective cohort study at primary healthcare clinics in Santiago, Chile.

**METHODS:**

The population was taken from the “Cito-Expert” database of 2010. The data were then organized according to the cytological and histological follow-up results of 2,547 women with inadequate cervical cytological reports over the 12-month period. The samples were assigned to groups based on the cause of inadequacy (smears with endocervical cells alone; insufficient, hemorrhagic, inflammatory or poorly fixed samples; insufficient and hemorrhagic samples; or insufficient and inflammatory specimens). The data were analyzed using the “conditional probability tree diagram” and descriptive statistics.

**RESULT:**

Half of the women (n = 1,285) met the requirements of the Ministry of Health for repeating these inadequate smears, and 1,104 of these women had normal cytological results (85.9%). The detection rate for cervical lesions according to group ranged from 0% (smears with endocervical cells alone or insufficient and hemorrhagic specimens) to 4.1% (poor fixation).

**CONCLUSION:**

The large proportion of normal results justifies revision of the current clinical guidelines. The results showed that it is not necessary to repeat the Pap test early on, with the exception of inadequate hemorrhagic and inflammatory cytological results.

## RESUMO

**CONTEXTO E OBJETIVO:**

Esfregaços inadequados de Papanicolaou não fornecem amostra satisfatória de células para avaliação, ficando assim mais difícil detectar anomalias citológicas cervicais. O objetivo deste estudo foi determinar os resultados de seguimento citológico e histológico de mulheres com relatórios de esfregaços inadequados nas unidades básicas de saúde em Santiago, Chile, 2010-2011.

**DESENHO E LOCAL:**

Estudo prospectivo de coorte em unidades básicas de saúde em Santiago, Chile.

**MÉTODOS:**

A população foi selecionada do banco de dados Cito-Expert em 2010. Na sequência, os dados foram organizados pelos resultados de seguimento citológico e histológico de 2.547 mulheres com relatórios inadequados de citologia cervical pelo período de 12 meses. As amostras foram atribuídas a grupos com base na causa de inadequação (esfregaços com células endocervicais; amostras insuficientes, inflamatórias, ou com má fixação, amostras insuficientes e hemorrágicas, ou espécimes insuficientes e inflamatórios). Os dados foram analisados com base no “conditional probability tree diagram” e estatísticas descritivas.

**RESULTADOS:**

Metade das mulheres (n = 1.285) preencheram os requisitos do Ministério da Saúde para repetir esses esfregaços; dessas mulheres, 1.104 tiveram resultados normais para citologia (85,9%). A detecção de lesões cervicais por grupo variou de 0% (esfregaços com apenas células endocervicais e espécimes insuficientes e hemorrágicos) para 4,1% (má fixação).

**CONCLUSÃO:**

Uma grande proporção de resultados normais justifica a revisão das diretrizes clínicas atuais. Os resultados mostraram que não é necessário repetir tão cedo o teste de Papanicolaou, exceto em resultados inadequados de hemorragia e citologia inflamatória.

## INTRODUCTION

Inadequate Pap smears are those that do not provide satisfactory cell samples for evaluation, thus making it more difficult to detect cervical cytological abnormalities and making it necessary to repeat the test, according to the current requirements of the National Cervical Cancer Research and Control Program in Chile.^1^^,^^2^ Trained professionals (midwives or gynecologists) are involved in this process. They are guided by regulations that establish uniform criteria for the patient’s prior clinical condition and for applying the conventional technique for collecting cervical cytology smears.^1^ The diagnostic codes of the Chilean National Program are used for reporting, and they are equivalent to the 2001 Bethesda System.^3^ These codes define the adequacy of cytological smears in categories that are considered to be satisfactory or unsatisfactory for evaluation. “Adequate samples” that are satisfactory for evaluation are classified as G8. “Inadequate samples” and their specific causes are classified between G0 and G7 and from G9 onwards.^2^

At the public health level, the relevance of inadequate Pap smears has been mentioned by Gonzalez et al., who stated that one of the main problems in the National Cervical Cancer Screening Program (NCCSP) in Mexico was inadequacy of cytological sampling.^4^ In this regard, Cendales et al. concluded that the quality of the cytological investigation is an important factor that can explain the low impact of the healthcare program on cervical cancer mortality in Colombia.^5^ Similarly, a report by Amaral et al. in Brazil suggested that inadequate cytological specimens might contribute towards increased numbers of undiagnosed precursor lesions, thus affecting the morbidity and mortality rates of cervical cancer.^6^ On the other hand, Adams et al. followed up cervical cytological smears, and the results indicated there was no difference between unsatisfactory and satisfactory smears with regard to the prevalence of the cervical lesions that were detected.^7^ These results were similar to those mentioned by Elumir-Tanner in their review.^8^ These findings challenge the notion that the Pap cytological test needs to be repeated after a period of 2-4 months, i.e. the practice suggested by the American Society for Colposcopy and Cervical Pathology, a standard that has been adopted by the Ministry of Health in Chile.^1^^,^^9^

## OBJECTIVE

The objective of this study was to determine the cytohistological follow-up results from women attended within primary healthcare centers in the Southern Metropolitan Area of Santiago, Chile, in 2010-2011, whose cytological reports presented inadequacies.

## METHODS

This was a descriptive, prospective, observational and quantitative epidemiological study based on following up cohorts of women with inadequate Pap test results that were reported within the National Cervical Cancer Research and Control Program in Chile.

In 2010, 47,541 exfoliative cervical cytological smears were collected using the conventional Pap sampling technique by professionals at primary healthcare (PHC) centers in the southern metropolitan area of Santiago, Chile. These smears were processed and classified in accordance with the national nomenclature, by the pathology department’s cytology laboratory at the Barros Luco Healthcare Complex, Santiago, Chile. The cytology reports were registered in the “Cito-Expert” national database. From this, all the inadequate Pap results from the 2010 period were selected, giving a total of 2,779 tests. This number corresponded to 5.84% of all the tests performed over the study period (2010). Evaluation of these inadequate specimens did not allow a positive or a negative cytological diagnostic description (Table 1).

**Table 1.**
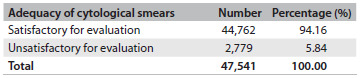
Distribution of exfoliative cervical cytological smears according to the adequacy of the sample, processed in the cytology laboratory at the Barros Luco Healthcare Complex, Santiago, Chile, 2010

Next, the women with inadequate results were selected, leaving a total population of 2,547. This population of women (n = 2,547) did not correspond to the number of tests (2,779), because some of the women had two inadequate Pap results.

The women were then organized into cohorts according to the cause of the inadequate result: G0 (smears with endocervical cells alone), 4 women; G1 (insufficient sample), 375 women; G2 (hemorrhagic cells), 249 women; G3 (inflammatory cells), 1,427 women; G4 (poor fixation), 61 women; G5 (insufficient and hemorrhagic samples), 40 women; G6 (insufficient and inflammatory samples), 391 women; G7 (endocervical and/or metaplasmic cells are not observed); and G9 (broken slide), none (Table 2). While preserving the participants’ anonymity, the information variables were the women’s ages at the time when the inadequate Pap smear was identified, the number and type of cytological and histological results and the length of follow-up (months), which ended with the diagnostic confirmation. This information was taken from the secondary source of the Cito-Expert cytological database.

**Table 2.**
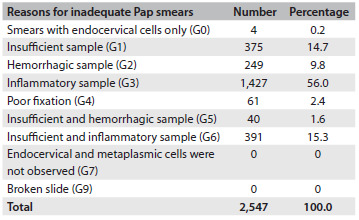
Distribution of women with inadequate cervical smear reports attended at primary healthcare centers in the southern metropolitan area of Santiago, Chile, 2010

The current requirements indicate that when an inadequate cytological result is obtained, the Pap test should be repeated after a three-month period. In the case of a normal Pap result, the test should be repeated after a three-year period, and when the results are pathological, the woman should be referred to the Cervical Pathology Unit. In the case of the present study, our follow-up contained all of the cytohistological tests performed on the seven cohorts over a twelve-month period, with the purpose of determining the significance of inadequate results.

Loss of follow-up was defined as absence of cytological or histological tests over the 12-month period. In this study, 1,262 women (49.5%) were lost from follow-up visits. These women did not undergo repetition of the inadequate Pap test at the end of the three-month period that was recommended by the Ministry of Health.

For data analysis, an Excel template was used. Descriptive statistics were calculated: mean, minimum and maximum, and relative frequency. A tracking tree, the “conditional probability tree diagram”, was used to identify the number, type, result, and time interval between the tests on these patients. The first row symbolized the first inadequate result. The second, third and fourth rows were the tests taken after the inadequate result.

## RESULTS

The women’s mean age was 39.1 years, with a minimum age of 17 years and a maximum of 84 years. The Cervical Cancer Program covers the group of women aged 25 to 64 years, which included 2,361 women (92.7%).

The requirement, set by the Ministry of Health, for repetition of smears due to inadequate cytological data for determining a diagnostic conclusion, was applicable to 50.5% of the women (n = 1,285). The other 49.5% of the women did not undergo repetition of smear collection because of personal or technical reasons (n = 1,262).

The re-administered cytological tests yielded the following results: a cytological report of cervical atypia was given for 8 women (0.6%); the repeated smear was also inadequate in the cases of 139 women (10.9%); a cervical lesion was detected in 34 women (2.6%); and normal results were reported in 1,104 women (85.9%).

**Figure 1.**
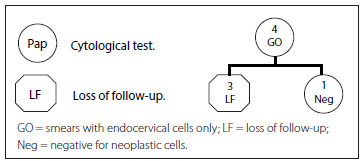
Follow-up on women with a G0 smear report (2010-2011).

Analysis on the inadequate results based on the specific cause was performed at the time of the follow-up visit of the cohorts of women using the conditional probability tree diagram. In Figure 1, the cytohistological follow-up on the cohort G0 (smears with endocervical cells alone) is represented. The first row represents the total number of women with a G0 Pap result (4 women). The second row shows that 3 of the 4 women did not repeat the Pap (LF), and one woman had a normal cytological result (negative).

**Figure 2.**
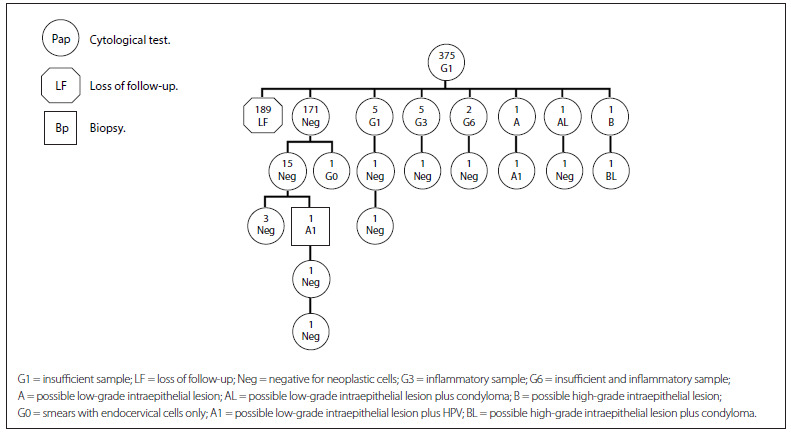
Follow-up on women with a G1 smear report (2010-2011).

Figure 2 presents the cytohistological follow-up on the cohort G1 (insufficient sample). The first row represents the total number of women with a G1 Pap result (375). The second row shows that the majority (189) of the 375 women did not comply with the ministerial rule for retesting (LF). Among those who did have the Pap repeated (186), a normal cytological report (negative) was the most frequent result (171), followed by 12 inadequate reports (G1, G3, G6) and 3 cases of lesions, of which two were low grade (A and AL) and one was high grade (B). The third row shows that the cases with lesions required further examination, thus confirming the results in two cases. This cohort showed that additional cytological specimens were collected from women with normal and inadequate results, a situation that does not fall within the regulations of the Ministry of Health.

**Figure 3.**
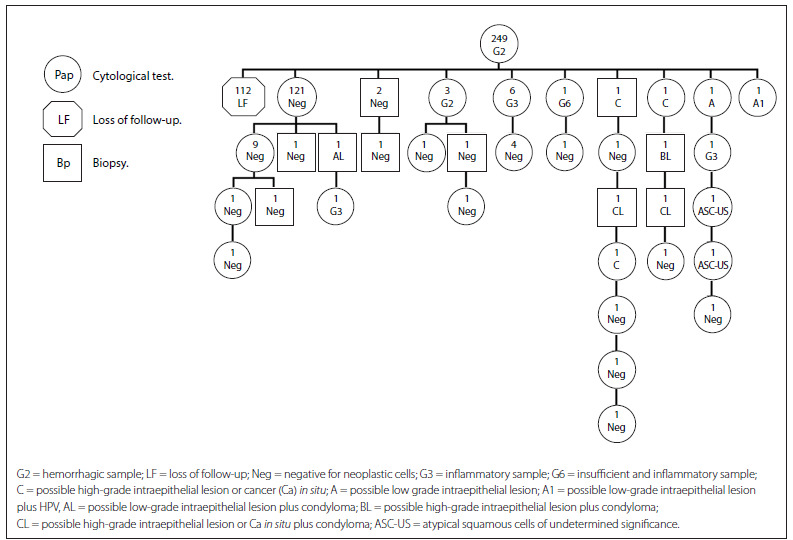
Follow-up on women with a G2 smear report (2010-2011).

Figure 3 presents the cytohistological follow-up on the cohort G2 (hemorrhagic samples). The first row represents the total number of women with a G2 Pap result (249). In the second row, the same trend as described in the previous cohorts was observed regarding the high proportion of women (112) who did not undergo a repeat cytological examination (LF). The follow-up tree shows that the most frequent outcome from the repeated examinations was a normal result (negative) (123 women). In the cases of 10 women, the inadequate results were repeated (G2, G3 and G6). There were four women (2.9%) with low (A and A1) and high-grade cervical lesions (C) with a cytological and histological follow-up for diagnosis and treatment that ultimately produced a normal cytological result. In the third row, additional tests were carried out for those with normal and inadequate cytological and histological results, which were most likely indicated for clinical reasons (Figure 3).

**Figure 4.**
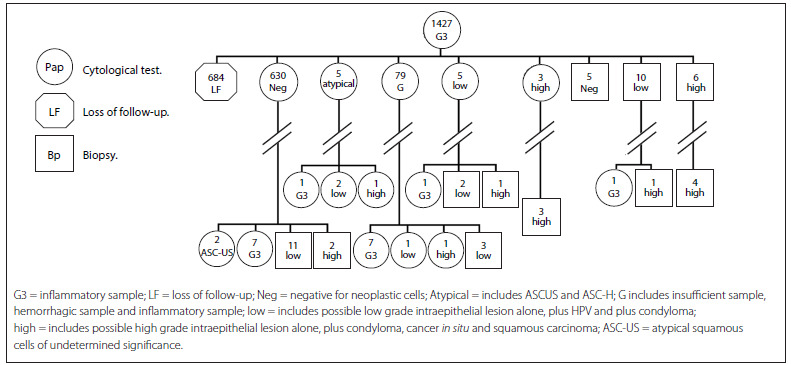
Follow-up on women with a G3 smear report (2010-2011).

Figure 4 presents the cytohistological follow-up on the cohort G3 (inflammatory samples). The first row represents the total number of women with a G3 Pap result (1,427). In the second row, 684 women were lost from the follow-up (LF) (47.9%). A normal result (negative) predominated among the cytological results (630 women), followed by 79 cases in which inadequate results (G) were obtained during the second Pap test. In eight patients, cervical lesions were detected through the cytological test. There were ten with low grade lesions (low), and six were detected by means of histology, corresponding to 3.2%. The // symbol represents a variety of diagnostic and treatment procedures. Finally, in the last row, 17 women still had inadequate reports (G3), 18 women were found to have low-grade lesions (low), and 13 were found to have high-grade lesions.

**Figure 5.**
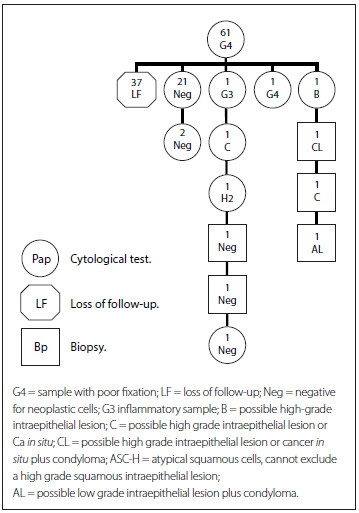
Follow-up on women with a G4 smear report (2010-2011).

Figure 5 presents the cytohistological follow-up on the cohort G4 (poor fixation). The first row represents the total number of women with a G4 Pap result (61). The second row shows that the majority (37) were lost from the follow-up (LF), while 24 women complied with the requirement to repeat the cytological test. From these, most had normal results (negative) (21 women), while two had inadequate results (G3 and G4) and one had a high-grade lesion (B) that required histological follow-up.

**Figure 6.**
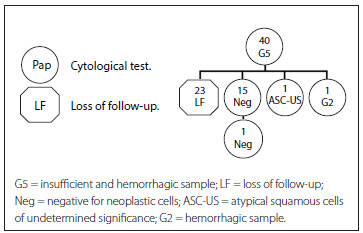
Follow-up on women with a G5 smear report (2010-2011).

Figure 6 presents the cohort G5 (insufficient and hemorrhagic samples). The first row represents the total number of women with a G5 Pap result (40). The second row shows that more than half did not repeat the test (LF) (23 women). Among the participants who re-took the test (17), most (15) had a normal result with no lesions.

**Figure 7.**
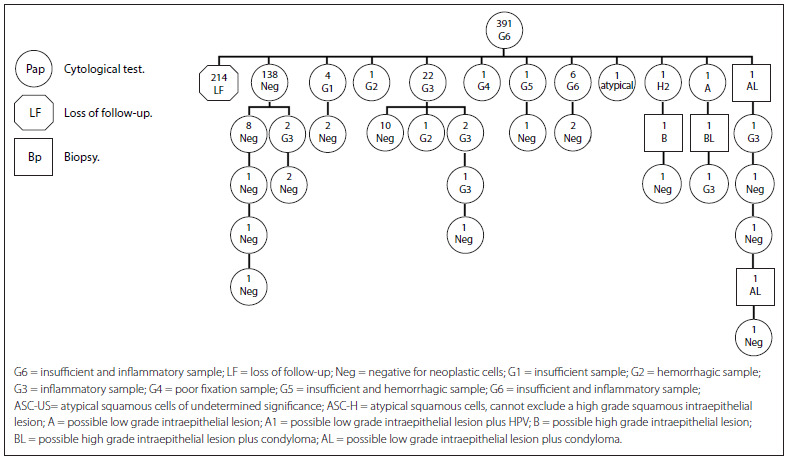
Follow-up on women with a G6 smear report (2010-2011).

Figure 7 presents the cohort G6 (insufficient and inflammatory samples). The first row represents the total number of women with a G6 Pap result (391 women). The second row shows that the majority of the women (214) did not repeat the test (54.7%) (LF). Among those who repeated the test, 138 had normal results (negative), 35 had inadequate results, (G1, G2, G3, G4, G5 and G6), two women had atypical results (atypical and H2) and two women had low-grade lesions. The third row shows that additional tests were carried out for those with normal results. Two women had a low-grade lesion (A and A1) and one had an atypical result (H2) that required cytohistological follow-up, which is represented in the subsequent rows.

During the process of obtaining a conclusive diagnosis, a total of 4,169 tests were performed, which included 4,066 smears and 103 biopsies, resulting in an average of 1.6 tests per woman.

There were 254 tests that had to be repeated and were not justified by the regulations of the Ministry of Health, i.e. those with normal results and those that had an inadequate result for the second time.

The probability of observing a cervical lesion with a repeat test according to the ministerial rule ranged from 1.1% (G6) to 4.1%. Cohorts G2, G3 and G4 showed the highest probabilities of lesions (2.9%, 3.2% and 4.1%, respectively). The G0 and G5 cohorts did not show any cytopathological lesions.

Our results relating to each cohort show that compliance with the ministerial rule allowed women with cervical lesions to have access to healthcare in accordance with the clinical protocol, which enabled them to have a good outcome for their pathological condition, especially women with inadequate results due to hemorrhagic or inflammatory samples.

## DISCUSSION

In our study on the outcomes from inadequate smears, three important findings were identified that relate to the following: i) low compliance with the ministerial regulation with regard to repetition of inadequate smears (one in two women); ii) high detection of normality in repeated reports from women who provided inadequate smears (nine out of ten); and iii) increased probability of lesions in women whose first smear was inadequate for hemorrhagic and inflammatory reasons.

We found that compliance with the recommendation for repetition of inadequate smears was deficient. This was similar to the findings from another study reported by the Cervical Cancer Screening Program in Ontario, Canada, in which only 35% of the women who had an inadequate Pap test repeated it within the recommended period of four months.^9^

Our study provides evidence that in nine out of ten cases, an inadequate result will be normal after the repetition of the test. Other studies have also reported similar findings. Adams et al. in the United States conducted a case-control study on inadequate and satisfactory smears, based on the Bethesda 2001 criteria, with a follow-up from 1998 to 2004 (including 167 patients with inadequate Pap tests and 350 with normal results). No significant differences were found between the adequate and inadequate smears with regard to the prevalence of cervical lesion detection.^7^ Furthermore, in a study evaluating a cervical cancer screening program in South Africa using a controlled clinical essay with patients recruited from 2003 to 2005, the incidence of cervical cytological abnormalities in women with previous normal smears was 6.48%, and it was 11.71% among those with inadequate smears. It was concluded that the incidence of high-grade lesions was low after repeating a normal or inadequate smear two years later.^10^ In the Netherlands, through a controlled clinical trial using data from the NETHCON clinical database of women from the Cervical Cancer Screening Program, the clinical relevance of an unsatisfactory Pap test was evaluated with a cytohistological follow-up period of 18 months. It was found that 0.7 and 0.8% of the samples from liquid-based and conventional cervical smear collections presented high-grade lesions.^11^ Different findings were obtained in a prospective study conducted in Norway over the period 1995-2001. The authors concluded, after two years of follow-up, that the risk of detection of high-grade lesions in women with an unsatisfactory Pap test was 1.6 to 4.0 times higher than in women with a normal Pap.^12^

Considering the existing scientific data as well as the current study, it is possible to justify the need for revision of the current clinical guidelines in Chile regarding repetition of inadequate cytological tests after a three-month period. Furthermore, as Gavranovic et al. showed, repetition of inadequate smears adds economic and social costs to the healthcare program without significantly increasing the lesion detection rate.^13^

This need for revision is already being considered on in the United States, where the American Society for Colposcopy and Cervical Pathology began the process of revising the 2006 clinical guidelines on inadequate handling of Pap tests, at a meeting that took place on September 14 and 15, 2012.^14^

## CONCLUSIONS

In 2010, 5.84% of Papanicolaou tests (2,779/47,541) resulted in inadequate smears in the southern metropolitan area of Santiago, Chile.

50.5% of these women (n = 1,285) had a repeat test after an inadequate smear, but only 23.6% (304/1,285) complied with the ministerial regulation, i.e. they repeated the test within a three-month period.

After obtaining the second Pap test, it was found that 85.9% of the women had normal smears (1,104/1,285); 0.6% obtained cytological reports of cervical atypia (n = 8 women); 10.9% had a repeat test after an inadequate smear (n = 139 women); 1.6% had a low-grade cervical lesion (n = 21 women); and 1.0% had a high-grade lesion (n = 13 women).

The results from this study justify revision of the current clinical guidelines in Chile regarding repetition of tests that provide inadequate cervical cytological data for any of the reasons considered within a three-month period. It is important to address the causes of inadequate smears and to repeat the test only in cases of hemorrhagic and inflammatory cytological results.
